# Relationship between gender and survival in a real-life cohort of patients with COPD

**DOI:** 10.1186/s12931-019-1154-3

**Published:** 2019-08-22

**Authors:** Maeva Zysman, Pierre-Régis Burgel, Isabelle Court-Fortune, Graziella Brinchault-Rabin, Pascale Nesme-Meyer, Pascale Surpas, Gaetan Deslée, Thierry Perez, Olivier Le Rouzic, Gilles Jebrak, Pascal Chanez, Jean-Louis Paillasseur, Denis Caillaud, Nicolas Roche, on behalf of the Initiatives BPCO scientific committee and investigators, P. R. Burgel, G. Deslee, P. Surpas, O. Le Rouzic, T. Perez, N. Roche, G. Brinchault-Rabin, D. Caillaud, P. Chanez, I. Court-Fortune, R. Escamilla, G. Jebrak, P. Nesme-Meyer, M. Zysman, C. Pinet, Brigitte Risse

**Affiliations:** 1 Pulmonary Department, Nancy, France; 2 Inserm U955, team 04, IMRB, Créteil, France; 3 Respiratory and Intensive Care Medicine Department, Cochin Hospital, AP-HP and Paris Descartes University (EA 2511), Sorbonne Paris Cité, Paris, France; 4 Service de Pneumologie, CHU Saint Etienne, Saint Etienne, France; 5 Service de Pneumologie, Hôpital Pontchaillou, Rennes, France; 6 Service de Pneumologie, Hôpital de la Croix-Rousse, Lyon, France; 7 Centre médical de Bayère, 30, route du Vieux-Château, 69380 Charnay, France; 8 Pulmonary Department, Maison Blanche University Hospital, INSERM U01250, Reims, France; 9 Univ.Lille, CNRS, Inserm, CHU Lille, Institut Pasteur de Lille, U1019 - UMR 8204 - CIIL - Center for Infection and Immunity of Lille, F-59000 Lille, France; 10 Service de Pneumologie, Hôpital Bichat, AP-HP, Paris, France; 11 Département des Maladies Respiratoires, AP-HM, Université de la Méditerranée, Marseille, France; 12 EFFI-STAT, Paris, France; 13 Service de Pneumologie, Hôpital Gabriel Montpied, CHU Clermont-Ferrand, Clermont-Ferrand, France; 14 Maéva Zysman 8 rue du général sarrail, 94000 Creteil, France

**Keywords:** Chronic obstructive pulmonary disease, Survival, Gender differences

## Abstract

**Background:**

Although COPD affects both men and women, its prevalence is increasing more rapidly in women. Disease outcomes appear different among women with more frequent dyspnea and anxiety or depression but whether this translates into a different prognosis remains to be determined. Our aim was to assess whether the greater clinical impact of COPD in women was associated with differences in 3-year mortality rates.

**Methods:**

In the French Initiatives BPCO real-world cohort, 177 women were matched up to 458 menon age (within 5-year intervals) and FEV_1_ (within 5% predicted intervals). 3-year mortality rate and survival were analyzed. Univariate and multivariate logistic regression analyses were performed.

**Results:**

For a given age and level of airflow obstruction, women with COPD had more severe dyspnea, lower BMI, and were more likely to exhibit anxiety. Nevertheless, three-year mortality rate was comparable among men and women, respectively 11.2 and 10.8%. In a multivariate model, the only factors significantly associated with mortality were dyspnea and malnutrition but not gender.

**Conclusion:**

Although women with COPD experience higher levels of dyspnea and anxiety than men at comparable levels of age and FEV1, these differences do not translate into variations in 3-year mortality rates.

**Trial registration:**

04–479.

## Background

Influence of gender on COPD expression and outcomes is an area of sustained interest [1, 2]. Although COPD affects both men and women, its prevalence is increasing more rapidly in women, particularly in younger women [1]*.* Women are more likely to be misdiagnosed [3], whereas there is increasing evidence suggesting gender-related differences in COPD risk. For example, female smokers are at greater risk of airflow obstruction than male smokers [4]. Disease progression and outcomes appear different among women and men with COPD [5, 6]. Younger women with COPD have a greater likelihood of more severe dyspnea and airflow limitation, and exhibit a higher risk of exacerbations [7, 8]. In COPD populations, several longitudinal studies showed an association between higher levels of symptoms and poorer prognosis [9]. Of interest, studies have found discrepant results regarding the relationship between gender and survival [10].

As shown in other studies [10, 11], previous analysis of the Initiatives BPCO cohort found that women suffer from higher levels of dyspnea and anxiety even after matching on age and FEV_1_ [12]. Whether these gender-related differences in symptoms translate into differences in survival remains unknown. Our aim was to assess whether the greater clinical impact of COPD in women was associated with differences in 3-year mortality rates.

## Methods

As previously described, Initiatives BPCO is a rolling cohort of patients with COPD followed at French University Hospitals [13]. The primary aim of the cohort was to study COPD phenotypes, as previously reported [13]. The following data are collected as part of routine practice at inclusion: demographic and anthropometric characteristics, occupational exposures, smoking history, chronic bronchitis, exacerbation frequency, dyspnea assessed by mMRC dyspnea scale, health status, physician diagnosed comorbidities (asthma, rhinitis, cardiovascular diseases, obesity, diabetes, mechanical limitation, psychological status), medications and post-bronchodilator spirometry (FEV_1_, FVC).

Men and women were matched up to 3:1 on age (within 5-year intervals) and FEV_1_ (within 5% predicted intervals) leading to a small loss of sample size. Three-year mortality rate and survival were analyzed using logistic regression and Kaplan-Meier analysis with log-rank test, respectively. Univariate comparisons between matched men and women were performed by chi2 and t-test. To identify which risk factors play a critical role as determinants of mortality in the studied population, we performed a multivariate stepwise logistic regression analysis with the following tested covariates: cumulative smoking, chronic bronchitis, mMRC grade, FEV1% predicted, exacerbation history during the year prior to inclusion, allergic rhinitis, associated asthma, nutritional status, hypertension, ischemic heart disease, left heart failure, diabetes, sleep apnea syndrome and age. Data are provided as median [Q1 – Q3] or n (%), as appropriate.

The study was approved by the Ethics Committee of Versailles (France), trial registration #04–479, and all subjects provided informed written consent.

## Results

**Fig. 1:**
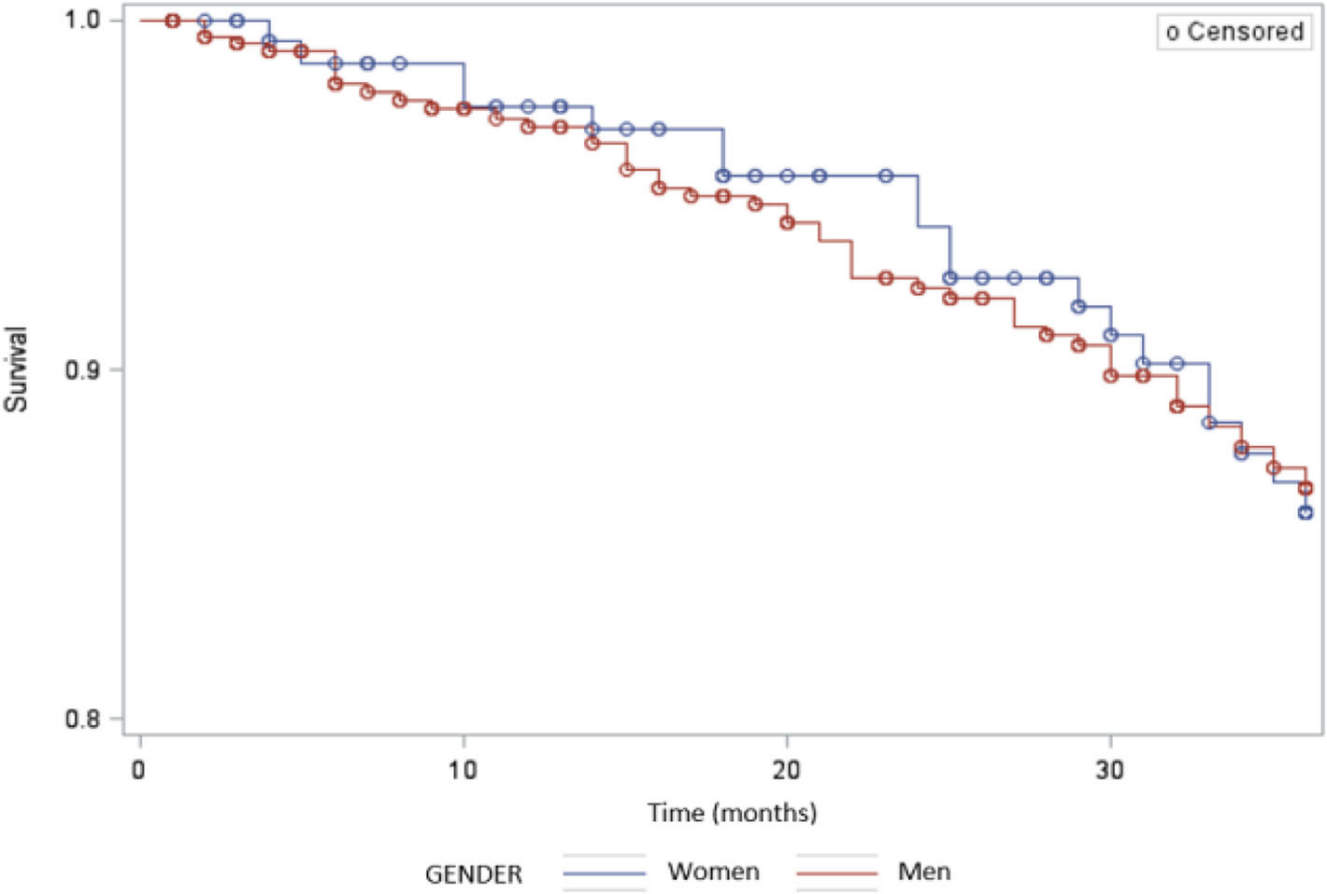
Survival according to gender, Kaplan Meier analysis, women in blue, men in red

Among 954 patients (226 women) with COPD included at the time of the analyses, 177 women were matched to 458 men. Unmatched (non-included) women did not differ from matched (included) ones except for age and FEV_1,_ which were the matching criteria (*data not shown*). Median values of age and percent predicted (pp) FEV_1_ were 63 years and 53%, respectively. Women had lower body mass index (BMI), higher mMRC dyspnea grade, resulting in a higher BOD (BMI, airflow obstruction, dyspnea) index, and a greater proportion of anxiety (defined by a hospital anxiety-depression-A subscore≥10). Rhinitis was more frequent in women, while coronary heart disease and obstructive sleep apnea syndrome were less frequent in women (Table 1). Three-year mortality rates were 11.2% in men and 10.8% in women with no significant difference (OR for men vs. women 0.9; 95% confidence interval [0.5–1.7]). Age at death was 68 years in men and 72 years in women with no significant difference. Survival was also comparable (Log-rank *p* = 0.9724, Fig. 1). In multivariate analysis, mortality was independently associated with only malnutrition (*p* = 0.02) and mMRC (*p* = 0.03), with cumulative smoking being retained in the model although of borderline significance (*p* = 0.06). Conversely, gender was not retained (*p* = 0.68).

**Table 1:** Characteristics of the studied population and univariate comparisons between age- and FEV1-matched (3:1 ratio) men (*n* = 458) and women (*n* = 177)

Variables	Women	Men	p
N	177	458	
Age (years)	62 [56–70]	63 [57–71]	0.4171
BMI	23.2 [20.2–27.1]	25.5 [22.1–29.0]	**< 0.0001**
Malnutrition (BMI < 18 kg/m2)	25 (14.1%)	31 (6.8%)	**0.003**
Smoking (Pack-years)	38.0 [24.8–57.0]	41 [27–56]	0.4080
FEV_1_ (%)	54 [37–69]	53 [36–67]	0.4257
Number of moderate to severe exacerbations in the previous year	1 [0–3]	1 [0–2]	0.1209
mMRC	2 [1–3]	1 [1–2]	**0.0014**
mMRC ≥ 2	100 (56.5%)	212 (46.3%)	0.021
BOD index	3 [1–4]	2 [1–4]	0.0831
SGRQ	46 [32–57] ^a^	43 [28–59] ^a^	0.5776
Asthma history	28 (15.8%)	52 (11.4%)	0.128
Rhinitis	29 (16.4%)	42 (9.2%)	**0.010**
Chronic bronchitis	118 (66.7%)	302 (65.9%)	0.862
Hypertension	59 (33.3%)	159 (34.7%)	0.742
Left heart failure	16 (9%)	54 (11.8%)	0.321
Ischemic heart disease	11 (6.2%)	79 (17.2%)	**0.0001**
Diabetes mellitus	16 (9%)	58 (12.7%)	0.202
Obstructive sleep apnea	4 (2.3%)	40 (8.7%)	**0.004**
HAD total score	15 [11–21] ^b^	12 [8–17] ^b^	**< 0.0001**
Anxiety HAD A ≥ 10	65 (44.5%)^b^	92 (27.6%)^b^	**0.0001**
Depression HAD D ≥ 10	34 (23.6%)^b^	59 (17.7%)^b^	0.136
3-year mortality	19 (10.8%)	51 (11.2%)	0.896
Age at death	72 [66–79]	68 [63–76]	0.1691

*BMI* body mass index, *HAD* hospital anxiety and depression scale, *mMRC* modified medical respiratory council, *SGRQ* Saint George’s Respiratory Questionnaire. Data are provided as median [Q1 – Q3] or n (%), as appropriate

^a^Missing data for SGRQ, *n* = 28 in women, *n* = 116 in men

^b^Missing data for HAD scores: *n* = 33 in women, *n* = 131 in men

## Discussion

Several studies have been performed to assess gender-related differences in COPD expression and many found more severe manifestations of the disease in women [6, 10]. Some studies suggest that women with chronic bronchitis have significantly worse survival [14] whereas others have demonstrated that survival does not vary among men and women in smaller cohorts [15]. In a previous analysis of the Initiatives BPCO cohort, for a given age and level of airflow obstruction, women with COPD had higher BOD (BMI, airflow obstruction, dyspnea) scores due to greater dyspnea and lower BMI, suggesting the possibility of worse prognosis in women. However, the present data showed no difference in survival between men and women matched for age and ppFEV1, both in univariate analyses. Furthermore, even after multivariate analyses confirming a link between worse prognosis, malnutrition and breathlessness, in accordance to BODE, gender was neither validated. Similar findings were reported in the TORCH study, in which the risk of death was similar among men and women once analyses were adjusted for differences in baseline confounders [10]. Previous data comparing 265 women and 272 men with COPD matched using BODE score have shown that all-cause mortality was higher in males than females. However, women and men were not matched on age and women were significantly younger (63 vs 67 years, *p* < 0.001) [16]. Even if this age difference is small, it can have an influence on mortality. To exclude this bias, men and women were matched on age in our study.

One limitation of this study is the relatively short-term survival analysis and the absence of available data regarding specific causes of mortality, which prevents from analyzing whether some specific mortality rates differ between men and women. Our results suggest that differences between men and women in prognostic scores (here, the BOD score) and burden of symptoms (exacerbation number and dyspnea) do not translate into higher mortality in women. Finally, as in other studies [2, 6, 12], male gender was associated with a more frequent history of cardiovascular disease (here, ischemic heart disease), maybe explaining why the risk of mortality is similar among men and women despite women exhibiting more symptoms.

## Conclusion

In the present study, COPD expression differed between men and women, with women experiencing more dyspnea and anxiety, and less diagnosed coronary heart disease and sleep apnea. These differences did not translate into significant differences in 3-year mortality rates and survival.

## Data Availability

The datasets used and analyzed during the current study are available from the corresponding author on reasonable request.

## Abbreviations

BMI: Body mass index
COPD: Chronic obstructive pulmonary disease
FEV1: Forced Expiratory volume in one second
GOLD: Global initiative for obstructive lung disease
HAD: Hospital anxiety and depression scale
mMRC: Modified Medical Research Council
OSAS: Obstructive sleep apnea syndrome
